# Supplementary material for: Cytokine Expression by Human Macrophage-Like Cells Derived from the Monocytic Cell Line THP-1 Differs between Treatment with Milk from Preterm- and Term-Delivering Mothers and Pasteurized Donor Milk
Source: Molecules. 2020 May 20;25(10):2376. doi: 10.3390/molecules25102376 (PMC7287623; doi:10.3390/molecules25102376)
Supplement: Supplementary file 1 [file molecules-25-02376-s001.pdf]

molecules

MDPI

# Supplementary Materials

**Table 1.** Sequences of the primers used for real-time RT-PCR.

| Target | Primer | Sequence 5′–3′ |
|---|---|---|
| Human TGF-α | FOR-TGF-α | CCCCAGGGACCTCTCTCTAATC |
| | REV-TGF-α | GGTTTGCTACAACATGGGCTACA |
| Human IL-6 | FOR-IL-6 | CATGGTGGATGCCGTTCA |
| | REV-IL-6 | TTTTCTGCCAGTGCCTCTTT |
| Human IL-12 | FOR-IL-12 | CATGGTGGATGCCGTTCA |
| | REV-IL-12 | ACCTCCACCTGCCGAGAAT |
| Human IL-10 | FOR-IL-10 | GCTGGAGGACTTTAAGGGTTACCT |
| | REV-IL-10 | CTTGATGTCTGGGTCTTGGTTCT |
| Human GAPDH | FOR-GAPDH | CGAGATCCCTCCAAAATCAA |
| | REV-GAPDH | TTCACACCCATGACGAACAT |
